# Supplementary material for: Acute Colon Inflammation Triggers Primary Motor Cortex Glial Activation, Neuroinflammation, Neuronal Hyperexcitability, and Motor Coordination Deficits
Source: Int J Mol Sci. 2022 May 11;23(10):5347. doi: 10.3390/ijms23105347 (PMC9141031; doi:10.3390/ijms23105347)
Supplement: Supplementary file 1 [file ijms-23-05347-s001.zip › ijms-1651794-supplementary.pdf]

Supplementary Material

Acute Colon Inflammation Triggers Primary Motor Cortex Glial Activation, Neuroinflammation, Neuronal Hyperexcitability, and Motor Coordination Deficits

Table S1. Effects of DSS-induced acute colon inflammation on body weight, muscular stretch, and grooming.

Parameters	Control	DSS
Body weight gain	100 ± 4.6	82.9 ± 3.7 *
Muscular stretch (s/g of body weight)	0.56 ± 0.1	0.44 ± 0.1
Grooming (number of movements)	4.28 ± 1.34	4.66 ± 0.84

Body weight gain was calculated as percentage with respect to the weight at the beginning of the treatment and respect to control rats. Muscular stretch was evaluated by using the hanging wire test to exhibit sustained limb tension to oppose their gravitational force and expressed by the time, in seconds, that an animal was able to hold itself per gram of body weight. Grooming was assessed by using the open field test as described in Methods. Data are presented as mean ± standard error of the mean. The number of animals was 6 for each experimental condition, (n = 6 for each group). Student' t-test: * $p < 0.05$, DSS-treated vs. control rats.
